# Supplementary material for: Effect on Chest Compression Fraction of Continuous Manual Compressions with Asynchronous Ventilations Using an i-gel® versus 30:2 Approach during Simulated Out-of-Hospital Cardiac Arrest: Protocol for a Manikin Multicenter Randomized Controlled Trial
Source: Healthcare (Basel). 2021 Mar 20;9(3):354. doi: 10.3390/healthcare9030354 (PMC8003743; doi:10.3390/healthcare9030354)
Supplement: Supplementary file 1 [file healthcare-09-00354-s001.zip › Supplementary File S2_English translation of the email sent to potential participants.pdf]

Dear colleague,

We need you!

As part of a research project, we are looking for pre-hospital healthcare workers from different horizons to take part in a multi-centre, randomised and controlled clinical trial on the initial management of cardiorespiratory arrest. We deliberately remain rather evasive on the content, but are confident in the results that this could generate and the substantial improvement in ambulance care autonomy that could result.

Participation will be in teams of two, randomly generated. The total time per team will be 1 hour and will be divided into three 20-minute workshops.

More information will be given on the day of the study and you will have the opportunity to ask any outstanding questions.

If you want to defend paramedic skills and participate in the recognition of the profession, don't hesitate any longer and take part in our study!

On behalf the investigators,

David Thurre and Loric Stuby
