# Supplementary material for: Effect on Chest Compression Fraction of Continuous Manual Compressions with Asynchronous Ventilations Using an i-gel® versus 30:2 Approach during Simulated Out-of-Hospital Cardiac Arrest: Protocol for a Manikin Multicenter Randomized Controlled Trial
Source: Healthcare (Basel). 2021 Mar 20;9(3):354. doi: 10.3390/healthcare9030354 (PMC8003743; doi:10.3390/healthcare9030354)
Supplement: Supplementary file 1 [file healthcare-09-00354-s001.zip › Supplementary File S3_Standard Operating Procedure (French version).pdf]

Standard Operating Procedure (French version)

Indications et prérequis

□ Confirme l'indication à l'i-gel : ACR
□ Aspiration fonctionnelle à portée de main

Préparation du matériel

□ I-GEL de taille appropriée
□ Lubrifiant non siliconé (type K-Y)
□ Moyen de fixation (lacet, scotch, fixe-tube)
□ Capteur EtCO2

Mise en place du dispositif

□ Lubrifie suffisamment l'i-gel (sauf partie antérieure)
□ Compressions thoraciques continues dès le début et durant toute la pose du dispositif
□ Vérifie l'absence de corps étranger dans la bouche avant l'insertion
□ Ouvre la bouche en effectuant une bascule de la tête et/ou une traction de la mandibule
□ Effectue l'insertion de l'i-gel sans forcer (se positionne de lui-même avec précision et naturellement par-dessus la structure laryngée)
□ Connecte le ballon auto-remplisseur avec le filtre suivi du capteur $EtCO_2$

Evaluation

□ Vérifie le bon placement du dispositif à l'aide de la capnographie, le corrige au besoin
□ S'assure du bon fonctionnement du système et de l'efficacité de la ventilation

Suite de soins

□ Effectue le MCE en continu sauf si ventilation inadéquate en l'absence de pauses
□ Optionnel : sécurise l'i-gel à l'aide d'un moyen de fixation
□ Optionnel : pose une sonde gastrique de taille adaptée et s'assure de sa position

Adaptations période COVID :

- l'équipement de protection des intervenants doit être maximal : au minimum masque FFP2, gants et lunettes de protection
- le filtre est monté directement sur l'i-gel lors de sa préparation
- le reste de la procédure est inchangé
