# Supplementary material for: Effect on Chest Compression Fraction of Continuous Manual Compressions with Asynchronous Ventilations Using an i-gel® versus 30:2 Approach during Simulated Out-of-Hospital Cardiac Arrest: Protocol for a Manikin Multicenter Randomized Controlled Trial
Source: Healthcare (Basel). 2021 Mar 20;9(3):354. doi: 10.3390/healthcare9030354 (PMC8003743; doi:10.3390/healthcare9030354)
Supplement: Supplementary file 1 [file healthcare-09-00354-s001.zip › Supplementary File S4_Standard Operating Procedure (English version).pdf]

Standard Operating Procedure (English version)

Indications and prerequisites

□ Confirms the i-gel® indication: cardiac arrest
□ Functional suction at hand

Preparation of material

□ Appropriately sized I-GEL®
□ Non-silicone lubricant (type K-Y)
□ Means of attachment (lace, tape, tube holder)
□ EtCO2 sensor

Device insertion

□ Sufficiently lubricates the i-gel® (except the front part)
□ Continuous chest compressions from the start and during all device insertion
□ Check for the absence of foreign bodies in the mouth before insertion
□ Open the mouth by tilting the head and / or pulling the mandible
□ Performs the insertion of the i-gel® without forcing (positions itself precisely and naturally over the laryngeal structure)
□ Connect the resuscitator with the filter and the EtCO2 sensor

Assessment

□ Checks the correct placement of the device using capnography, correct it if necessary
□ Ensures the proper functioning of the system and the efficiency of the ventilation

Care suite

□ Performs continuous MCE unless ventilation is inadequate in the absence of interruptions
□ Optional: secure the i-gel® using a means of attachment
□ Optional: place a gastric tube of the appropriate size and ensure its position

COVID period adaptations:

- the protective equipment of the providers must be maximum: at least N95 mask, gloves and eyes protection
- the filter is placed directly on the i-gel® during its preparation
- the rest of the procedure is unchanged
