# Supplementary material for: Effect on Chest Compression Fraction of Continuous Manual Compressions with Asynchronous Ventilations Using an i-gel® versus 30:2 Approach during Simulated Out-of-Hospital Cardiac Arrest: Protocol for a Manikin Multicenter Randomized Controlled Trial
Source: Healthcare (Basel). 2021 Mar 20;9(3):354. doi: 10.3390/healthcare9030354 (PMC8003743; doi:10.3390/healthcare9030354)
Supplement: Supplementary file 1 [file healthcare-09-00354-s001.zip › Supplementary File S7_PHP code.pdf]

DISPLAY RESULTS

```
<?php

// Ouvre une session
session_start();

// Connexion à mysql
include("connect.php");


?>
<!DOCTYPE html>
<html>

<head>
        <meta http-equiv="Content-Type" content="text/html; charset=UTF-8" />

        <meta name="title" content="Etude CPR / IGEL" />
        <meta name="description" content="résultats">
        <link rel="stylesheet"
href="https://maxcdn.bootstrapcdn.com/bootstrap/4.0.0/css/bootstrap.min.css"
integrity="sha384-Gn5384xqQ1aoWXA+058RXPxPg6fy4IWvTNh0E263XmFcJlSAwiGgFAW/dAiS6J
Xm" crossorigin="anonymous">
</head>

<body>
<?php

// Converti les secondes en hh mm ss
function millisecondsToTime($milliseconds) {
        $seconds = $milliseconds/1000;
    $dtF = new \DateTime('@0');
    $dtT = new \DateTime("@$seconds");
    return $dtF->diff($dtT)->format('%i min, %s sec');
}

// Affiche aux 2 min
$IncrementTime = $_POST['IncrementTime_choix'];
// Temps total
$TotalTimeSession = $_POST['TotalTimeSession_choix'];
// Nom de la table
$table_name = $_POST['table_name_choix'];

echo '<b>Table : '.$table_name.' / Temps de session : '.$TotalTimeSession.' ms
('.millisecondsToTime($TotalTimeSession).') / Echelle de temps :
'.$IncrementTime.' ms ('.millisecondsToTime($IncrementTime).')</b><p><p>';

// Initialisation des données
$new_time_cpr = 0 ;
$last_time_cpr = 0 ;
$count_cpr_inactivity = 0 ;
$count_cpr_inactivity_total = 0 ;
$time_cpr_inactivity = 0 ;
```

```
$time_cpr_inactivity_total = 0 ;
$first_cpr = 0 ;
$first_vent = 0 ;
$first_vent_inefficace = 0 ;
$first_shock = 0 ;
$count_shock = 0;
$time_shock = 0 ;
$time_shock_moyen = 0 ;
$time_shock_total = 0 ;
$last_time_shock = 0 ;
$last_time_intermediaire = 0 ;
$total_time_total = 0 ;
$ventil_volume_moyen = 0 ;
$ventil_volume_moyen_total = 0 ;
$count_ventil_strong = 0 ;
$count_ventil_strong_total = 0 ;
$count_ventil_weak = 0 ;
$count_ventil_weak_total = 0 ;
$count_ventil = 0 ;
$count_ventil_total = 0 ;
$compDepth_moyen = 0 ;
$compDepth_moyen_total = 0 ;
$count_mce = 0 ;
$count_mce_total = 0 ;
$count_compDepth_weak = 0 ;
$count_compDepth_weak_total = 0 ;
$count_compDepth_strong = 0 ;
$count_compDepth_strong_total = 0 ;
$compReleaseDepth_moyen = 0 ;
$compReleaseDepth_moyen_total = 0 ;
$count_compReleaseDepth = 0 ;
$count_compReleaseDepth_total = 0 ;
$count_compMeanRate_slow = 0 ;
$count_compMeanRate_slow_total = 0 ;
$count_compMeanRate_fast = 0 ;
$count_compMeanRate_fast_total = 0 ;
$compMeanRate_moyen = 0 ;
$compMeanRate_moyen_total = 0 ;
$time_intermediaire = 0 ;

// Recherche liste
$query_cpr='SELECT
        type, msecs, type2, value
FROM
        '.$table_name.'
WHERE
        (type = "CprCompEvent" OR type = "CprVentEvent" OR type =
"ShockDelivered") AND (type2 = "endTime" OR type2 = "shockEnergy")
ORDER BY msecs';

$result_cpr=$mysqli->query($query_cpr);

while ($myrow_cpr = mysqli_fetch_row($result_cpr)) {
```

```
                        // Coupe la session à 10 min depuis le 1er massage
                        if ($total_time_total<=$TotalTimeSession) {

                                        // Définit le temps ZERO du 1er massage
                                        if ($myrow_cpr[0] == 'CprCompEvent') {
                                                        if ($first_cpr==0) {
$first_cpr = $myrow_cpr[1]; }
                                        }

                                        // Attribue le temps du massage suivant
                                        if ($myrow_cpr[0] == 'CprCompEvent') {

                                                        $new_time_cpr =
$myrow_cpr[1]-$first_cpr;
                                                        $count_mce=$count_mce+1;

                                                        // Recherche la
profondeur du massage
                                                        $query_mce_check='SELECT
                                                                value
                                                        FROM
                                                                '.$table_name.'
                                                        WHERE
                                                                type =
"CprCompEvent" AND type2 = "compDepth" AND msecs = "'.$myrow_cpr[1].'"
                                                        ORDER BY msecs';


$result_mce_check=$mysqli->query($query_mce_check);

$myrow_mce_chech=mysqli_fetch_row($result_mce_check);


                                                        // Calcul les
compressions
                                                        $compDepth_moyen =
$compDepth_moyen+$myrow_mce_chech[0];

                                                        // Si compression trop
faible
                                                        if ($myrow_mce_chech[0]
< 50) { $count_compDepth_weak = $count_compDepth_weak+1; }
                                                        // Si compression trop
forte
                                                        if ($myrow_mce_chech[0]
> 60) { $count_compDepth_strong = $count_compDepth_strong+1; }

                                                        // Recherche le
relâchement du thorax
                                                        $query_mce_check='SELECT
                                                                value
                                                        FROM
                                                                '.$table_name.'
                                                        WHERE
```

```
                                                                type =
"CprCompEvent" AND type2 = "compReleaseDepth" AND msecs = "'.$myrow_cpr[1].'"
                                                        ORDER BY msecs';


$result_mce_check=$mysqli->query($query_mce_check);

$myrow_mce_chech=mysqli_fetch_row($result_mce_check);


                                                        // Calcul les
relâchements
                                                        $compReleaseDepth_moyen
= $compReleaseDepth_moyen+$myrow_mce_chech[0];

                                                        // Si pas assez de
relâchement
                                                        if ($myrow_mce_chech[0]
> 5) { $count_compReleaseDepth = $count_compReleaseDepth+1; }

                                                        // Recherche la
fréquence du massage
                                                        $query_mce_check='SELECT
                                                                value
                                                        FROM
                                                                '.$table_name.'
                                                        WHERE
                                                                type =
"CprCompEvent" AND type2 = "compMeanRate" AND msecs = "'.$myrow_cpr[1].'"
                                                        ORDER BY msecs';


$result_mce_check=$mysqli->query($query_mce_check);

$myrow_mce_chech=mysqli_fetch_row($result_mce_check);


                                                        // Calcul les fréquences
                                                        $compMeanRate_moyen =
$compMeanRate_moyen+$myrow_mce_chech[0];

                                                        // Si compression trop
faible
                                                        if ($myrow_mce_chech[0]
< 100) { $count_compMeanRate_slow = $count_compMeanRate_slow+1; }
                                                        // Si compression trop
forte
                                                        if ($myrow_mce_chech[0]
> 120) { $count_compMeanRate_fast= $count_compMeanRate_fast+1; }

                                        }

                                        // Recherche les ventilations
                                        if ($myrow_cpr[0] == 'CprVentEvent') {
```

```
                                                        // Recherche si
ventilation efficace

$query_vent_chech='SELECT
                                                                value
                                                        FROM
                                                                '.$table_name.'
                                                        WHERE
                                                                type =
"CprVentEvent" AND type2 = "ventVolume" AND msecs = "'.$myrow_cpr[1].'"
                                                        ORDER BY msecs';


$result_vent_chech=$mysqli->query($query_vent_chech);
                                                        $myrow_vent_chech =
mysqli_fetch_row($result_vent_chech);

                                                        // Recherche si volume
>= 300 ml
                                                        if ($myrow_vent_chech[0]
>= 300) {


$new_time_vent = $myrow_cpr[1];
                                                                        if
($first_vent==0) { $first_vent = $myrow_cpr[1]; }

$affiche_volume_vent = '<font color="#0000FF">'.$myrow_vent_chech[0].'
ml</font>';
                                                        } else {

$new_time_vent_inefficace = $myrow_cpr[1];
                                                                        if
($first_vent_inefficace==0) { $first_vent_inefficace = $myrow_cpr[1]; }

$affiche_volume_vent = '<font color="#800080">'.$myrow_vent_chech[0].' ml !!! (<
300 ml)</font>'; }

                                                        // Calcul les volumes

$ventil_volume_moyen=$ventil_volume_moyen+$myrow_vent_chech[0];

$count_ventil=$count_ventil+1;
                                                        // Si volume trop faible
                                                        if ($myrow_vent_chech[0]
< 300) { $count_ventil_weak = $count_ventil_weak+1; }
                                                        // Si volume trop fort
                                                        if ($myrow_vent_chech[0]
> 700) { $count_ventil_strong = $count_ventil_strong+1; }
                                        }

                                        // Attribue temps du 1er choc et
additionne les temps des chocs et les compte
```

```
                                        if ($myrow_cpr[0] == 'ShockDelivered') {



                                                        if ($first_shock==0) {
                                                                $first_shock =
$myrow_cpr[1];
                                                                $last_time_shock
= $myrow_cpr[1];
                                                        }

                                                        // Compte les chocs

$count_shock=$count_shock+1;

$count_shock2[$count_shock] = $count_shock;

                                                        // Mémorise le temps de
chaque choc
                                                        $time_shock =
$myrow_cpr[1]-$first_cpr;

$time_shock2[$count_shock] = $time_shock;

                                                        // Calcule le temps
entre chaque choc
                                                        $time_between_shock =
$myrow_cpr[1] - $last_time_shock;

$time_between_shock2[$count_shock] = $time_between_shock;

                                                        // Calcul le temps total
depuis le 1er choc
                                                        $time_shock_total =
$myrow_cpr[1] - $first_shock;

$time_shock_total2[$count_shock] = $time_shock_total ;

                                                        // Mémorise le temps du
choc précédent
                                                        $last_time_shock =
$myrow_cpr[1];
                                        }

                                        // Affiche que si demandé dans
formulaire
                                        if (!isset($_POST['Affiche_resume'])) {
                                                        // Affiche tous les
temps
                                                        if
($myrow_cpr[0]!='CprCompEvent' AND $myrow_cpr[0]!='CprVentEvent' AND
$myrow_cpr[0]!='ShockDelivered') { echo '<strike>'; }
                                                        if
($myrow_cpr[0]=='CprVentEvent') { echo '<font color=blue>'; }
```

```
                                                            if
($myrow_cpr[0]=='ShockDelivered') { echo '<font color=#FF8C00>'; }
                                                            if ($first_cpr>0) {
                                                                            echo
$myrow_cpr[0].' - '.($myrow_cpr[1]-$first_cpr).' ms /
'.(($myrow_cpr[1]-$first_cpr)/1000).' s / ';

$time_event = ($myrow_cpr[1]-$first_cpr)/1000/60;
                                                                            echo
number_format($time_event, 2, '.', '').'<br>';

                                                            }
                                                            if
($myrow_cpr[0]!='CprCompEvent' AND $myrow_cpr[0]!='CprVentEvent' AND
$myrow_cpr[0]!='ShockDelivered') { echo '</strike>'; }
                                                            if
($myrow_cpr[0]=='CprVentEvent') {
                                                                            echo
'</font>';
                                                                            echo
'<b>'.$affiche_volume_vent.'</b><br>';
                                                            }
                                                            if
($myrow_cpr[0]=='ShockDelivered') { echo '</font>'; }
                                            }


                                            // Additionne si le temps entre 2
massages est supérieur à 1000 ms
                                            if ((($last_time_cpr+1000) <
$new_time_cpr) AND ($first_cpr > 0)) {

                                                            $delta_cpr_inactivity =
$new_time_cpr-$last_time_cpr;
                                                            if
(!isset($_POST['Affiche_resume'])) { echo '<font color="#FF0000">Delta
'.$delta_cpr_inactivity.' ms / '.($delta_cpr_inactivity/1000).' s<br>'; }

$time_cpr_inactivity=$time_cpr_inactivity+$delta_cpr_inactivity;
                                                            if
(!isset($_POST['Affiche_resume'])) { echo 'Total : <b>'.$time_cpr_inactivity.'
ms / '.($time_cpr_inactivity/1000).' s</b></font><br>'; }
                                            }

                                            // Redéfinit les variables
                                            if ($myrow_cpr[0] == 'CprCompEvent') {
                                                            $last_time_cpr =
$myrow_cpr[1]-$first_cpr;
                                            }
                                            $total_time_total=$last_time_cpr;

                                            // Mémorise les temps intermédiaires
                                            if
($time_intermediaire<=$TotalTimeSession AND
```

```
$total_time_total>=$time_intermediaire+$IncrementTime) {

                                                        $last_time_intermediaire
= $total_time_total - $last_time_intermediaire;

$time_intermediaire2[$time_intermediaire] = $last_time_intermediaire;
                                                        $last_time_intermediaire
= $total_time_total;

$total_time2[$time_intermediaire] = $total_time_total;


$ventil_volume_moyen2[$time_intermediaire] = $ventil_volume_moyen;

$ventil_volume_moyen_total = $ventil_volume_moyen_total + $ventil_volume_moyen;
                                                        $ventil_volume_moyen =
0;


$count_ventil2[$time_intermediaire] = $count_ventil;
                                                        $count_ventil_total =
$count_ventil_total + $count_ventil;
                                                        $count_ventil = 0 ;


$count_ventil_strong2[$time_intermediaire] = $count_ventil_strong;

$count_ventil_strong_total = $count_ventil_strong_total + $count_ventil_strong;
                                                        $count_ventil_strong =
0;


$count_ventil_weak2[$time_intermediaire] = $count_ventil_weak;
                                                        $count_ventil_weak_total
= $count_ventil_weak_total + $count_ventil_weak;
                                                        $count_ventil_weak = 0;


$compDepth_moyen2[$time_intermediaire] = $compDepth_moyen;
                                                        $compDepth_moyen_total =
$compDepth_moyen_total + $compDepth_moyen;
                                                        $compDepth_moyen = 0;


$count_mce2[$time_intermediaire] = $count_mce;
                                                        $count_mce_total =
$count_mce_total + $count_mce;
                                                        $count_mce = 0;


$count_compDepth_weak2[$time_intermediaire] = $count_compDepth_weak;

$count_compDepth_weak_total = $count_compDepth_weak_total +
```

```
$count_compDepth_weak;
                                                        $count_compDepth_weak =
0;


$count_compDepth_strong2[$time_intermediaire] = $count_compDepth_strong;

$count_compDepth_strong_total = $count_compDepth_strong_total +
$count_compDepth_strong;
                                                        $count_compDepth_strong
= 0;


$count_compReleaseDepth2[$time_intermediaire] = $count_compReleaseDepth;

$count_compReleaseDepth_total = $count_compReleaseDepth_total +
$count_compReleaseDepth;
                                                        $count_compReleaseDepth
= 0;


$compReleaseDepth_moyen2[$time_intermediaire] = $compReleaseDepth_moyen;

$compReleaseDepth_moyen_total = $compReleaseDepth_moyen_total +
$compReleaseDepth_moyen;
                                                        $compReleaseDepth_moyen
= 0;


$compMeanRate_moyen2[$time_intermediaire] = $compMeanRate_moyen;

$compMeanRate_moyen_total = $compMeanRate_moyen_total + $compMeanRate_moyen;
                                                        $compMeanRate_moyen = 0;


$count_compMeanRate_slow2[$time_intermediaire] = $count_compMeanRate_slow;

$count_compMeanRate_slow_total = $count_compMeanRate_slow_total +
$count_compMeanRate_slow;
                                                        $count_compMeanRate_slow
= 0;


$count_compMeanRate_fast2[$time_intermediaire] = $count_compMeanRate_fast;

$count_compMeanRate_fast_total = $count_compMeanRate_fast_total +
$count_compMeanRate_fast;
                                                        $count_compMeanRate_fast
= 0;


$time_cpr_inactivity2[$time_intermediaire] = $time_cpr_inactivity;

$time_cpr_inactivity_total = $time_cpr_inactivity_total + $time_cpr_inactivity;
```

```
                                                        $time_cpr_inactivity =
0;


$time_intermediaire=$time_intermediaire+$IncrementTime;

                                        }

                        }

}
?><p>

<table  class="table table-sm">
  <thead class="thead-dark">
    <tr>
      <th scope="col">Temps</th>
      <th scope="col">Temps<br>session<br>(depuis le 1er MCE)</th>
          <th scope="col"><font color="#FF8C00">Temps entre 1er MCE<br>et 1er
choc</th>
          <th scope="col"><font color="#FF8C00">Stats choc</th>
      <th scope="col"><font color="#0000FF">Temps entre 1er MCE<br>et 1ère
ventilation (>300 ml)</th>
          <th scope="col"><font color="#0000FF">Stats ventilation</th>
          <th scope="col"><font color="#FF0000">Stats massage</th>
      <th scope="col"><font color="#FF0000">Total temps NO FLOW<br>(si pause > 1
seconde)</font><br></th>
    </tr>
  </thead>
  <tbody>
        <?php
                // initialisation compteur
                $count_round=0;

                // Affiche les temps intermédiaires
                for ($time_increase = $IncrementTime; $time_increase <=
$TotalTimeSession; $time_increase=$time_increase+$IncrementTime) {
                        // Cherche le time d'avant pour afficher les bonnes
données
                        $time_intermediaire=$time_increase-$IncrementTime;

                        // Incrémente le compteur
                        $count_round = $count_round + 1;
                        ?>
                        <tr>
                          <th scope="row"><?php echo '<nobr>->
'.($time_increase/1000/60).' min</nobr>'; ?></th>
                          <td><?php
                                        echo
$time_intermediaire2[$time_intermediaire].'
ms<br>'.($time_intermediaire2[$time_intermediaire]/1000).' s<br>';
                                        $time_min =
$time_intermediaire2[$time_intermediaire]/1000/60;
                                        echo number_format($time_min, 2, '.',
```

```
'').' min<br>'; ?></td>
                          <td><font color="#FF8C00"><?php
                                        // Affiche que si dans la tranche de
temps correspondante
                                        if
($first_shock<$total_time2[$time_intermediaire] AND
$first_shock>($total_time2[$time_intermediaire]-$IncrementTime)) {
                                                        echo
($first_shock-$first_cpr).' ms<br>'.(($first_shock-$first_cpr)/1000).' s<br>';
                                                        $time_min =
($first_shock-$first_cpr)/1000/60;
                                                        echo
number_format($time_min, 2, '.', '').' min<br>';
                                        } ?></font></td>
                          <td><font color="#FF8C00"><?php

                                        // Affiche les temps intermédiaires
                                        for ($nb_increase_shock = 1 ;
$nb_increase_shock <= $count_shock2[$count_shock];
$nb_increase_shock=$nb_increase_shock+1) {

                                                        // Affiche que si dans
la tranche de temps correspondante
                                                        if
(($time_shock2[$nb_increase_shock]<$total_time2[$time_intermediaire]) AND
($time_shock2[$nb_increase_shock]>($total_time2[$time_intermediaire]-$IncrementT
ime))) {
                                                                        echo
'<b>Choc '.$count_shock2[$nb_increase_shock].'</b><br>';
                                                                        echo
($time_shock2[$nb_increase_shock]).'
ms<br>'.(($time_shock2[$nb_increase_shock])/1000).' s<br>';

$time_min = ($time_shock2[$nb_increase_shock])/1000/60;
                                                                        echo
number_format($time_min, 2, '.', '').' min<br>';
                                                                        if
(isset($last_shock_time)) {

echo 'Temps depuis dernier choc
:<br>'.(($time_between_shock2[$nb_increase_shock])/1000).' s<br>';

$time_shock_moyen = $time_shock_moyen+$time_between_shock2[$nb_increase_shock];

echo 'Temps moyen entre les chocs
:<br><b>'.number_format((($time_shock_moyen/($count_shock2[$nb_increase_shock]-1
))/1000), 2, '.', '').' s</b><br><br>';
                                                                        }

$last_shock_time = $time_shock2[$nb_increase_shock];
                                                        }

                                        }
```

```
                                ?></font></td>
                        <td><font color="#0000FF"><?php
                                // Affiche que si dans la tranche de
temps correspondante
                                if
(($first_vent-$first_cpr)<$total_time2[$time_intermediaire] AND
($first_vent-$first_cpr)>($total_time2[$time_intermediaire]-$IncrementTime)) {
                                                echo
($first_vent-$first_cpr).' ms<br>'.(($first_vent-$first_cpr)/1000).' s<br>';
                                                $time_min =
($first_vent-$first_cpr)/1000/60;
                                                echo
number_format($time_min, 2, '.', '').' min';
                                } ?></font><br>
                            <?php
                                // Affiche que si dans la tranche de
temps correspondante
                                if
(($first_vent_inefficace-$first_cpr)<$total_time2[$time_intermediaire] AND
($first_vent_inefficace-$first_cpr)>($total_time2[$time_intermediaire]-$Incremen
tTime)) {
                                                if
($first_vent_inefficace>0 AND !isset($affiche_once)) { ?><i><font
color="#800080">1ère ventilation insuffisante (<300 ml) :<br><?php
                                                                echo
($first_vent_inefficace-$first_cpr).'
ms<br>'.(($first_vent_inefficace-$first_cpr)/1000).' s<br>';

$time_min = ($first_vent_inefficace-$first_cpr)/1000/60;
                                                                echo
number_format($time_min, 2, '.', '').' min';
                                                                echo
'</i></font>';

$affiche_once=1;
                                                }
                                } ?></td>
                        <td><font color="#0000FF"><?php
                                echo
'<b>'.$count_ventil2[$time_intermediaire].' ventilations</b><br> pour un volume
moyen de
'.number_format(($ventil_volume_moyen2[$time_intermediaire]/$count_ventil2[$time
_intermediaire]), 2, '.', '').' ml<br>';
                                echo 'FV :
'.number_format(($count_ventil2[$time_intermediaire]/($time_intermediaire2[$time
_intermediaire]/1000/60)), 2, '.', '').'/min<br>'; ?>
                                <font color="#800080"><?php
                                        echo
$count_ventil_weak2[$time_intermediaire].' ventilations < 300 ml (';
                                        echo
number_format((($count_ventil_weak2[$time_intermediaire]/$count_ventil2[$time_in
termediaire])*100), 2, '.', '').'%)<br>';
                                        echo
$count_ventil_strong2[$time_intermediaire].' ventilations > 700 ml (';
```

```
                                                echo
number_format((($count_ventil_strong2[$time_intermediaire]/$count_ventil2[$time_
intermediaire])*100), 2, '.', '').'%)<br>';
                                                echo 'Efficacité ventilation :
'.number_format((($count_ventil2[$time_intermediaire]-($count_ventil_strong2[$ti
me_intermediaire]+$count_ventil_weak2[$time_intermediaire]))/$count_ventil2[$tim
e_intermediaire]*100), 2, '.', '').'%</font>';
                                        ?>
                                        </font></td>
                            <td><font color="#000000"><?php
                                        echo
'<b>'.$count_mce2[$time_intermediaire].' massages</b> (FC moyenne :
'.number_format(($count_mce2[$time_intermediaire]/($time_intermediaire2[$time_in
termediaire]/1000/60)), 2, '.', '').'/min) : <br>';
                                        echo '<u>Profondeur moyenne</u> :
'.number_format(($compDepth_moyen2[$time_intermediaire]/$count_mce2[$time_interm
ediaire]), 2, '.', '').' mm<br>';
                                                echo
'<li>'.$count_compDepth_weak2[$time_intermediaire].' massages < 50 mm (';
                                                echo
number_format((($count_compDepth_weak2[$time_intermediaire]/$count_mce2[$time_in
termediaire])*100), 2, '.', '').'%)<br>';
                                                echo
'<li><b>'.($count_mce2[$time_intermediaire]-($count_compDepth_weak2[$time_interm
ediaire]+$count_compDepth_strong2[$time_intermediaire])).' massages entre 50 mm
et 60 mm (';
                                                echo
number_format(((($count_mce2[$time_intermediaire]-($count_compDepth_weak2[$time_
intermediaire]+$count_compDepth_strong2[$time_intermediaire]))/$count_mce2[$time
_intermediaire])*100), 2, '.', '').'%)</b><br>';
                                                echo
'<li>'.$count_compDepth_strong2[$time_intermediaire].' massages > 60 mm (';
                                                echo
number_format((($count_compDepth_strong2[$time_intermediaire]/$count_mce2[$time_
intermediaire])*100), 2, '.', '').'%)<br>';
                                        echo '<u>Relâchement moyen</u> :
'.number_format(($compReleaseDepth_moyen2[$time_intermediaire]/$count_mce2[$time
_intermediaire]), 2, '.', '').' mm<br>';
                                                echo
'<li>'.$count_compReleaseDepth2[$time_intermediaire].' relâchements insuffisants
(> 5 mm) (';
                                                echo
number_format((($count_compReleaseDepth2[$time_intermediaire]/$count_mce2[$time_
intermediaire])*100), 2, '.', '').'%)<br>';
                                                echo '<li>'.'<b>Efficacité
relâchement :
'.number_format((($count_mce2[$time_intermediaire]-$count_compReleaseDepth2[$tim
e_intermediaire])/$count_mce2[$time_intermediaire]*100), 2, '.',
'').'%</b><br>';
                                        echo '<u>Fréquence moyenne massages</u>
:
'.number_format(($compMeanRate_moyen2[$time_intermediaire]/$count_mce2[$time_int
ermediaire]), 2, '.', '').'/min<br>';
                                                echo
```

```
'<li>'.$count_compMeanRate_slow2[$time_intermediaire].' massages < 100/min (';
                                                echo
number_format((($count_compMeanRate_slow2[$time_intermediaire]/$count_mce2[$time
_intermediaire])*100), 2, '.', '').'%)<br>';
                                                echo
'<li><b>'.($count_mce2[$time_intermediaire]-($count_compMeanRate_slow2[$time_int
ermediaire]+$count_compMeanRate_fast2[$time_intermediaire])).' massages entre
100 et 120/min (';
                                                echo
number_format(((($count_mce2[$time_intermediaire]-($count_compMeanRate_slow2[$ti
me_intermediaire]+$count_compMeanRate_fast2[$time_intermediaire]))/$count_mce2[$
time_intermediaire])*100), 2, '.', '').'%)</b><br>';
                                                echo
'<li>'.$count_compMeanRate_fast2[$time_intermediaire].' massages > 120/min (';
                                                echo
number_format((($count_compMeanRate_fast2[$time_intermediaire]/$count_mce2[$time
_intermediaire])*100), 2, '.', '').'%)<br>';

                                                ?>
                                        </font></td>
                            <td><font color="#FF0000"><?php
                                        echo
$time_cpr_inactivity2[$time_intermediaire].'
ms<br>'.($time_cpr_inactivity2[$time_intermediaire]/1000).' s';

$total_percent=(($time_intermediaire2[$time_intermediaire]-$time_cpr_inactivity2
[$time_intermediaire])/$time_intermediaire2[$time_intermediaire])*100;
                                        echo '<br>% MCE vs No flow (CCF) :
<b>'.number_format($total_percent, 2, '.', '').'%';
                                        $ccf[$count_round] =
number_format($total_percent, 2, '.', '');
                                        ?></b></font></td>
                          </tr>
                          <?php
                } ?>
  </tbody>
</table>

<b>Totaux</b><br>

<table  class="table table-sm">
  <thead class="thead-dark">
    <tr>
      <th scope="col">Temps</th>
      <th scope="col">Temps<br>session<br>(depuis le 1er MCE)</th>
          <th scope="col"><font color="#FF8C00">Temps entre 1er MCE<br>et 1er
choc</th>
          <th scope="col"><font color="#FF8C00">Stats choc</th>
      <th scope="col"><font color="#0000FF">Temps entre 1er MCE<br>et 1ère
ventilation (>300 ml)</th>
          <th scope="col"><font color="#0000FF">Stats ventilation</th>
          <th scope="col"><font color="#FF0000">Stats massage</th>
      <th scope="col"><font color="#FF0000">Total temps NO FLOW<br>(si pause > 1
seconde)</font><br></th>
```

```
    </tr>
  </thead>
  <tbody>
        <tr>
          <th scope="row"><?php echo number_format(($total_time_total/1000/60), 2, '.', '').' min'; ?></th>
          <td><?php
                        echo $total_time_total.'
ms<br>'.($total_time_total/1000).' s<br>';
                        $time_min = $total_time_total/1000/60;
                        echo number_format($time_min, 2, '.', '').' min<br>';
?></td>
          <td><font color="#FF8C00"><?php
                        echo ($first_shock-$first_cpr).'
ms<br>'.(($first_shock-$first_cpr)/1000).' s<br>';
                        $time_min = ($first_shock-$first_cpr)/1000/60;
                        echo number_format($time_min, 2, '.', '').' min<br>';
                        ?></font></td>
          <td><font color="#FF8C00"><?php
                        echo '<b>'.$count_shock.' chocs</b><br>';
                        echo 'Temps moyen entre les chocs
:<br><b>'.number_format((($time_shock_moyen/($count_shock-1))/1000), 2, '.',
'').' s</b><br>';
                        ?></font></td>
          <td><font color="#0000FF"><?php
                        echo ($first_vent-$first_cpr).'
ms<br>'.(($first_vent-$first_cpr)/1000).' s<br>';
                        $time_min = ($first_vent-$first_cpr)/1000/60;
                        echo number_format($time_min, 2, '.', '').' min';
                        ?></font><br>
                  <?php
                        if ($first_vent_inefficace>0) {
                                ?><i><font color="#800080">1ère
ventilation insuffisante (<300 ml) :<br><?php
                                echo
($first_vent_inefficace-$first_cpr).'
ms<br>'.(($first_vent_inefficace-$first_cpr)/1000).' s<br>';
                                $time_min =
($first_vent_inefficace-$first_cpr)/1000/60;
                                echo number_format($time_min, 2, '.',
'').' min';
                                echo '</i></font>';
                        } ?></td>
          <td><font color="#0000FF"><?php
                        echo '<b>'.$count_ventil_total.' ventilations</b><br>
pour un volume moyen de
'.number_format(($ventil_volume_moyen_total/$count_ventil_total), 2, '.', '').'
ml<br>';
                        echo 'FV :
'.number_format(($count_ventil_total/($total_time_total/1000/60)), 2, '.',
'').'/min<br>'; ?>
                        <font color="#800080"><?php
                              echo $count_ventil_weak_total.' ventilations <
300 ml (';
```

```
                                echo
number_format((($count_ventil_weak_total/$count_ventil_total)*100), 2, '.',
'').'%)<br>';
                                echo $count_ventil_strong_total.' ventilations >
700 ml (';
                                echo
number_format((($count_ventil_strong_total/$count_ventil_total)*100), 2, '.',
'').'%)<br>';
                                echo 'Efficacité ventilation :
'.number_format((($count_ventil_total-($count_ventil_strong_total+$count_ventil_
weak_total))/$count_ventil_total*100), 2, '.', '').'%</font>';
                        ?>
                        </font></td>
          <td><font color="#000000"><?php
                        echo '<b>'.$count_mce_total.' massages</b> (FC moyenne :
'.number_format(($count_mce_total/($total_time_total/1000/60)), 2, '.',
'').'/min) : <br>';
                        echo '<u>Profondeur moyenne</u> :
'.number_format(($compDepth_moyen_total/$count_mce_total), 2, '.', '').'
mm<br>';
                                echo '<li>'.$count_compDepth_weak_total.'
massages < 50 mm (';
                                echo
number_format((($count_compDepth_weak_total/$count_mce_total)*100), 2, '.',
'').'%)<br>';
                                echo
'<li><b>'.($count_mce_total-($count_compDepth_weak_total+$count_compDepth_strong
_total)).' massages entre 50 mm et 60 mm (';
                                echo
number_format(((($count_mce_total-($count_compDepth_weak_total+$count_compDepth_
strong_total))/$count_mce_total)*100), 2, '.', '').'%)</b><br>';
                                echo '<li>'.$count_compDepth_strong_total.'
massages > 60 mm (';
                                echo
number_format((($count_compDepth_strong_total/$count_mce_total)*100), 2, '.',
'').'%)<br>';
                        echo '<u>Relâchement moyen</u> :
'.number_format(($compReleaseDepth_moyen_total/$count_mce_total), 2, '.', '').'
mm<br>';
                                echo '<li>'.$count_compReleaseDepth_total.'
relâchements insuffisants (> 5 mm) (';
                                echo
number_format((($count_compReleaseDepth_total/$count_mce_total)*100), 2, '.',
'').'%)<br>';
                                echo '<li>'.'<b>Efficacité relâchement :
'.number_format((($count_mce_total-$count_compReleaseDepth_total)/$count_mce_tot
al*100), 2, '.', '').'%</b><br>';
                        echo '<u>Fréquence moyenne massages</u> :
'.number_format(($compMeanRate_moyen_total/$count_mce_total), 2, '.',
'').'/min<br>';
                                echo '<li>'.$count_compMeanRate_slow_total.'
massages < 100/min (';
                                echo
number_format((($count_compMeanRate_slow_total/$count_mce_total)*100), 2, '.',
```

```
'').'%)<br>';
                                echo
'<li><b>'.($count_mce_total-($count_compMeanRate_slow_total+$count_compMeanRate_
fast_total)).' massages entre 100 et 120/min (';
                                echo
number_format(((($count_mce_total-($count_compMeanRate_slow_total+$count_compMea
nRate_fast_total))/$count_mce_total)*100), 2, '.', '').'%)</b><br>';
                                echo '<li>'.$count_compMeanRate_fast_total.'
massages > 120/min (';
                                echo
number_format((($count_compMeanRate_fast_total/$count_mce_total)*100), 2, '.',
'').'%)<br>';
                                ?>
                        </font></td>
          <td><font color="#FF0000"><?php
                        echo $time_cpr_inactivity_total.'
ms<br>'.($time_cpr_inactivity_total/1000).' s';

$total_percent=(($total_time_total-$time_cpr_inactivity_total)/$total_time_total
)*100;
                        echo '<br>% MCE vs No flow (CCF) :
<b>'.number_format($total_percent, 2, '.', '').'%';
                        // Valeur pour CSV file
                        $overall_CCF = number_format($total_percent, 2, '.',
'');
                        $CompDepth =
number_format(($compDepth_moyen_total/$count_mce_total), 2, '.', '');
                        $intarget_depth =
number_format(((($count_mce_total-($count_compDepth_weak_total+$count_compDepth_
strong_total))/$count_mce_total)*100), 2, '.', '');
                        $below_depth =
number_format((($count_compDepth_weak_total/$count_mce_total)*100), 2, '.', '');

                        $above_depth =
number_format((($count_compDepth_strong_total/$count_mce_total)*100), 2, '.',
'');
                        $CompRelease =
number_format((($count_mce_total-$count_compReleaseDepth_total)/$count_mce_total
*100), 2, '.', '');
                        $CompFrequency =
number_format(($compMeanRate_moyen_total/$count_mce_total), 2, '.', '');

                        $intarget_frequency =
number_format(((($count_mce_total-($count_compMeanRate_slow_total+$count_compMea
nRate_fast_total))/$count_mce_total)*100), 2, '.', '');
                        $below_frequency =
number_format((($count_compMeanRate_slow_total/$count_mce_total)*100), 2, '.',
'');
                        $above_frequency =
number_format((($count_compMeanRate_fast_total/$count_mce_total)*100), 2, '.',
'');
                        $time_shock_csv = (($first_shock-$first_cpr)/1000);
                        $Nb_shock = $count_shock;
                        $time_ventilation_csv = (($first_vent-$first_cpr)/1000);
```

```
                        $Nb_Vent = $count_ventil_total;
                        $VentVolume =
number_format(($ventil_volume_moyen_total/$count_ventil_total), 2, '.', '');
                        $intarget_volume =
number_format((($count_ventil_total-($count_ventil_strong_total+$count_ventil_we
ak_total))/$count_ventil_total*100), 2, '.', '');
                        $below_volume =
number_format((($count_ventil_weak_total/$count_ventil_total)*100), 2, '.', '');
                        $above_volume =
number_format((($count_ventil_strong_total/$count_ventil_total)*100), 2, '.',
'');

                        ?></b></font></td>
        </tr>
  </tbody>
</table>

<table border="0" cellspacing="0" bordercolor="#000000" width="100%" summary="">
        <tr>
                <form action="export_csv.php" name="affiche_result"
method="post" enctype="multipart/form-data">









                        <td align="center" valign="middle">
                                <input type="hidden" name="count_round"
value="<?php echo $count_round; ?>">
                                <input type="hidden" name="table_name"
value="<?php echo $table_name; ?>">
                                <input type="hidden" name="overall_CCF"
value="<?php echo $overall_CCF; ?>">
                                <?php
                                for ($nb_increase_round = 1 ; $nb_increase_round
<= $count_round; $nb_increase_round=$nb_increase_round+1) {
                                                ?>
                                                <input type="hidden"
name="CCF[<?php echo $nb_increase_round; ?>]" value="<?php echo
$ccf[$nb_increase_round]; ?>">
                                                <?php
                                }
                                ?>

                                <input type="hidden" name="CompDepth"
value="<?php echo $CompDepth; ?>">
                                <input type="hidden" name="intarget_depth"
value="<?php echo $intarget_depth; ?>">
                                <input type="hidden" name="below_depth"
```

```
value="<?php echo $below_depth; ?>">
                                <input type="hidden" name="above_depth"
value="<?php echo $above_depth; ?>">
                                <input type="hidden" name="CompRelease"
value="<?php echo $CompRelease; ?>">
                                <input type="hidden" name="CompFrequency"
value="<?php echo $CompFrequency; ?>">
                                <input type="hidden" name="intarget_frequency"
value="<?php echo $intarget_frequency; ?>">
                                <input type="hidden" name="below_frequency"
value="<?php echo $below_frequency; ?>">
                                <input type="hidden" name="above_frequency"
value="<?php echo $above_frequency; ?>">
                                <input type="hidden" name="time_shock_csv"
value="<?php echo $time_shock_csv; ?>">
                                <input type="hidden" name="Nb_shock"
value="<?php echo $Nb_shock; ?>">
                                <input type="hidden" name="time_ventilation_csv"
value="<?php echo $time_ventilation_csv; ?>">
                                <input type="hidden" name="Nb_Vent" value="<?php
echo $Nb_Vent; ?>">
                                <input type="hidden" name="VentVolume"
value="<?php echo $VentVolume; ?>">
                                <input type="hidden" name="intarget_volume"
value="<?php echo $intarget_volume; ?>">
                                <input type="hidden" name="below_volume"
value="<?php echo $below_volume; ?>">
                                <input type="hidden" name="above_volume"
value="<?php echo $above_volume; ?>">

                                <input type="radio" name="separation"
value="pointvirgule" checked style="font-size: 8pt;"> Point-virgule
;     
                                <input type="radio" name="separation"
value="virgule" style="font-size: 8pt;"> Virgule ,
                                <br>
                                <input type="submit" value="CSV"><p>


                        </td>






                </form>
        </tr>
        <tr>
                <form action="index.php" name="affiche_result" method="post"
enctype="multipart/form-data">
```

```
                                <td align="center" valign="middle">

                                        <input type="submit" value="RETOUR">

                                </td>

                        </form>
                </tr>
        </table>

        </body>
        </html>
```

INDEX

```
<?php

// Ouvre une session
session_start();

// Connexion à mysql
include("connect.php");


?>
<!DOCTYPE html>
<html>

<head>
        <meta http-equiv="Content-Type" content="text/html; charset=UTF-8" />

        <meta name="title" content="Etude CPR / IGEL" />
        <meta name="description" content="résultats">
        <link rel="stylesheet"
href="https://maxcdn.bootstrapcdn.com/bootstrap/4.0.0/css/bootstrap.min.css"
integrity="sha384-Gn5384xqQ1aoWXA+058RXPxPg6fy4IWvTNh0E263XmFcJlSAwiGgFAW/dAiS6J
Xm" crossorigin="anonymous">
</head>

<body>




<table border="0" cellspacing="0" width="100%" bordercolor="#000000" summary="">
         <tr>
                        <form action="affiche_result.php" name="affiche_result"
method="post" enctype="multipart/form-data">









                                <td align="left" valign="middle">
                                        <b>Choisir la simulation :</b><BR>

                                        <select name="table_name_choix">
                                                                <?php

                                                                // Recherche
liste
```

```
$query_list_tables='SHOW TABLES FROM ybch_simman_cpr';


$result_list_tables=$mysqli->query($query_list_tables);

                                                                 while
($myrow_list_tables = mysqli_fetch_row($result_list_tables)) {

?><option value="<?php echo $myrow_list_tables[0]; ?>"><?php echo
$myrow_list_tables[0]; ?></option>
                                                                         <?php
                                                                 }

                                                                 ?>

                                        </select><p>


                                </td>
                        </tr>
                        <tr>
                                <td align="left" valign="middle">
                                        <b>Temps de session (défaut : 10 min :
600 000 msec)</b><BR>
                                        <input type="text"
name="TotalTimeSession_choix" size="6" maxlength="6" value="600000"><p>
                                </td>
                        </tr>
                        <tr>
                                <td align="left" valign="middle">
                                        <b>Echelle de temps (défaut : 2 min :
120 000 msec)</b><BR>
                                        <input type="text"
name="IncrementTime_choix" size="6" maxlength="6" value="120000"><p>
                                </td>
                        </tr>
                        <tr>
                                <td align="left" valign="middle">
                                        <input type="checkbox"
name="Affiche_resume" value="oui"> Affiche que les résumés<p>
                                </td>
                        </tr>
                        <tr>
                                <td align="left" valign="middle">
```

```
                                        <input type="submit" value="Envoyer">


                                 </td>
                    </form>





               </tr>

       </table>

       </body>
       </html>
```

EXPORT IN CSV

```
<?php

// Ouvre une session
session_start();

// Définit le séparateur
if ($_POST['separation']=='virgule') { $separateur=','; } elseif
($_POST['separation']=='pointvirgule') { $separateur=';'; }

// Prépare les titres de la table
$output = '"ID"'.$separateur.'"overall_CCF"'.$separateur;
// Ajoute les titres selon le nombre de boucle définies à la page précédente
for ($nb_increase_round = 1 ; $nb_increase_round <= $_POST['count_round'];
$nb_increase_round=$nb_increase_round+1) {
                $output .= '"CCF'.$nb_increase_round.'"'.$separateur;
}
// Et ajoute le reste des titres
$output .=
'"CompDepth"'.$separateur.'"intarget_depth"'.$separateur.'"below_depth"'.$separa
teur.'"above_depth"'.$separateur.'"CompRelease"'.$separateur.'"CompFrequency"'.$
separateur.'"intarget_frequency"'.$separateur.'"below_frequency"'.$separateur.'"
above_frequency"'.$separateur.'"time_shock"'.$separateur.'"Nb_shock"'.$separateu
r.'"time_ventilation"'.$separateur.'"Nb_Vent"'.$separateur.'"VentVolume"'.$separ
ateur.'"intarget_volume"'.$separateur.'"below_volume"'.$separateur.'"above_volum
e"';
$output .="\n";

// Prépare les champs de la ligne de la table
$output .=
'"'.$_POST['table_name'].'"'.$separateur.'"'.$_POST['overall_CCF'].'"'.$separate
ur;
// Ajoute les champs selon le nombre de boucle définies à la page précédente
for ($nb_increase_round = 1 ; $nb_increase_round <= $_POST['count_round'];
$nb_increase_round=$nb_increase_round+1) {
                $output .=
'"'.$_POST['CCF'][$nb_increase_round].'"'.$separateur;
}
// Et ajoute le reste des champs
$output .=
'"'.$_POST['CompDepth'].'"'.$separateur.'"'.$_POST['intarget_depth'].'"'.$separa
teur.'"'.$_POST['below_depth'].'"'.$separateur.'"'.$_POST['above_depth'].'"'.$se
parateur.'"'.$_POST['CompRelease'].'"'.$separateur.'"'.$_POST['CompFrequency'].'
"'.$separateur.'"'.$_POST['intarget_frequency'].'"'.$separateur.'"'.$_POST['belo
w_frequency'].'"'.$separateur.'"'.$_POST['above_frequency'].'"'.$separateur.'"'.
$_POST['time_shock_csv'].'"'.$separateur.'"'.$_POST['Nb_shock'].'"'.$separateur.
'"'.$_POST['time_ventilation_csv'].'"'.$separateur.'"'.$_POST['Nb_Vent'].'"'.$se
parateur.'"'.$_POST['VentVolume'].'"'.$separateur.'"'.$_POST['intarget_volume'].
'"'.$separateur.'"'.$_POST['below_volume'].'"'.$separateur.'"'.$_POST['above_vol
ume'].'"';
$output .="\n";
```

```
/*
// Get The Field Name
for ($i = 0; $i < $columns_total; $i++) {
                // $heading = mysql_field_name($sql, $i);
                $heading = (mysqli_fetch_field_direct($sql, $i)->name);
                $output .= '"'.$heading.'",';
}
$output .="\n";

// Get Records from the table
while ($row = mysqli_fetch_array($sql)) {
                for ($i = 0; $i < $columns_total; $i++) {
                                $output .='"'.$row["$i"].'",';

                }
                // Enlève le point et l'espace
                $output .="\n";
}
*/

// Download the file
$filename = "cpr_".$_POST['table_name'].".csv";
header('Content-type: application/csv');
header('Content-Disposition: attachment; filename='.$filename);

echo $output;
exit;


?>
```
