# Supplementary material for: Effect on Chest Compression Fraction of Continuous Manual Compressions with Asynchronous Ventilations Using an i-gel® versus 30:2 Approach during Simulated Out-of-Hospital Cardiac Arrest: Protocol for a Manikin Multicenter Randomized Controlled Trial
Source: Healthcare (Basel). 2021 Mar 20;9(3):354. doi: 10.3390/healthcare9030354 (PMC8003743; doi:10.3390/healthcare9030354)
Supplement: Supplementary file 1 [file healthcare-09-00354-s001.zip › Supplementary File S9_Consent form (English version).pdf]

**RESEARCH**<br>**Introduction of the i-gel device in the early cardiac arrest management in a simulated situation**	
Responsible for the research project:	Stuby Loric<br>Registered Paramedic, Advanced Federal Diploma of Higher Education, CAS in Patient Oriented Clinical Research<br>Principal Investigator<br>l.stuby@gt-ambulances.ch

WRITTEN CONSENT

Consent to participate in research

Based on the above information, I confirm that I was duly informed about the study and that I was able to ask all the questions I had. Consequently, I agree to participate in the research "Introduction of the i-gel device in the early cardiac arrest management in a simulated situation", and I authorize:

- the use of data for scientific purposes and the publication of research results in scientific journals or books, it being understood that the data will remain anonymous and that no information will be given on my identity; ☐ YES ☐ NO

I voluntarily chose to participate in this research. I have been informed that I can withdraw at any time without providing any justification.

This consent does not release the organizers of the research from their responsibilities. I retain all my rights guaranteed by law.

First name, Last name ....

Signature ....

Date ....

RESEARCHER'S COMMITMENT

The information on this consent form and the responses I have given to the participant accurately describe the project.

I agree to conduct this study in accordance with good clinical trial practice.

I agree that the research participant will receive a copy of this consent form.

First name, Last name ....

Signature ....

Date ....
