# Supplementary material for: Effect on Chest Compression Fraction of Continuous Manual Compressions with Asynchronous Ventilations Using an i-gel® versus 30:2 Approach during Simulated Out-of-Hospital Cardiac Arrest: Protocol for a Manikin Multicenter Randomized Controlled Trial
Source: Healthcare (Basel). 2021 Mar 20;9(3):354. doi: 10.3390/healthcare9030354 (PMC8003743; doi:10.3390/healthcare9030354)
Supplement: Supplementary file 1 [file healthcare-09-00354-s001.zip › Supplementary Table S5_Demographics data questionnaire.pdf]

First questionnaire

French original version	English translated version
**Complété pré-scénario**	**Completed pre-scenario**
Identifiant de l'équipe (noté sur l'enveloppe)	*Team ID* (indicated on the envelope)
Âge (en années)	*Age* (in years)
Genre □ Femme □ Homme □ Autre	*Gender* □ Female □ Male □ Other
Années d'expériences professionnelles	*Years of professional experience*
Titre professionnel □ Ambulancier·ère ES □ Technicien·ne ambulancier·ère	*Professional title* □ Paramedic □ EMT
Nombre estimé d'insertions d'i-gel effectuées sur un mannequin au cours de l'année dernière	*Estimated number of i-gel insertion performed on manikin during last year*
Nombre estimé d'insertions d'i-gel effectuées sur un patient réel au cours de la dernière année	*Estimated number of i-gel insertion performed on real patient during the last year*
Rôle □ Leader □ Équipier	*Role* □ Team Leader □ Team Member
