# Supplementary material for: Effect on Chest Compression Fraction of Continuous Manual Compressions with Asynchronous Ventilations Using an i-gel® versus 30:2 Approach during Simulated Out-of-Hospital Cardiac Arrest: Protocol for a Manikin Multicenter Randomized Controlled Trial
Source: Healthcare (Basel). 2021 Mar 20;9(3):354. doi: 10.3390/healthcare9030354 (PMC8003743; doi:10.3390/healthcare9030354)
Supplement: Supplementary file 1 [file healthcare-09-00354-s001.zip › Supplementary Table S6_Assessment's questionnaire.pdf]

Second questionnaire

French original version	English translated version
**Complété durant le scénario**	**Completed during the scenario**
Identifiant de l'équipe (noté sur l'enveloppe)	*Team ID* (indicated on the envelope)
Tentative réussie □ 1 □ 2 □ 3 □ Échec	*Successful attempt* □ 1 □ 2 □ 3 □ Failure
**Complété post-scenario**	**Completed post-scenario**
Identifiant de l'équipe (noté sur l'enveloppe)	*Team ID* (indicated on the envelope)
Satisfaction concernant l'approche<br><br>□ Pas du tout satisfait<br><br>□ Pas satisfait<br><br>□ Neutre / indéterminé<br><br>□ Satisfait<br><br>□ Très satisfait	*Satisfaction regarding approach*<br><br>□ Not satisfied at all<br><br>□ Not satisfied<br><br>□ Neutral / undetermined<br><br>□ Satisfied<br><br>□ Very satisfied
Charge cognitive<br><br>En utilisant l'échelle de notation, veuillez évaluer la quantité d'effort mental nécessaire pour compléter ce scénario de simulation (entourez).	*Cognitive load*<br><br>Using the rating scale, please rate the amount of mental effort that was required to complete this simulation scenario (circle).
Très, très petite 1 2 3 4 5 6 7 8 9 Très, très haute	Very, very small 1 2 3 4 5 6 7 8 9 Very, very high
