# Supplementary material for: Effect on Chest Compression Fraction of Continuous Manual Compressions with Asynchronous Ventilations Using an i-gel® versus 30:2 Approach during Simulated Out-of-Hospital Cardiac Arrest: Protocol for a Manikin Multicenter Randomized Controlled Trial
Source: Healthcare (Basel). 2021 Mar 20;9(3):354. doi: 10.3390/healthcare9030354 (PMC8003743; doi:10.3390/healthcare9030354)
Supplement: Supplementary file 1 [file healthcare-09-00354-s001.zip › Supplementary File S8_Consent form (French version).pdf]

**RECHERCHE**<br>**Introduction du dispositif i-gel dans la gestion précoce de l'ACR en situation simulée**	
Responsable du projet de recherche :	Stuby Loric<br>Ambulancier diplômé ES, CAS en recherche clinique orientée patient<br>Investigateur Principal<br>l.stuby@gt-ambulances.ch

CONSENTEMENT ÉCRIT

Consentement de participation à la recherche

Sur la base des informations qui précèdent, je confirme avoir été dûment informé·e au sujet de l'étude et avoir pu poser toutes les questions que je me posais. En conséquence, je donne mon accord pour participer à la recherche « *Introduction du dispositif i-gel dans la gestion précoce de l'ACR en situation simulée* », et j'autorise :

- l'utilisation des données à des fins scientifiques et la publication des résultats de la recherche dans des revues ou livres scientifiques, étant entendu que les données resteront anonymes et qu'aucune information ne sera donnée sur mon identité ; ☐ OUI ☐ NON

J'ai choisi volontairement de participer à cette recherche. J'ai été informé-e du fait que je peux me retirer en tout temps sans fournir de justifications.

Ce consentement ne décharge pas les organisateurs de la recherche de leurs responsabilités. Je conserve tous mes droits garantis par la loi.

Prénom, Nom ....

Signature ....

Date ....

ENGAGEMENT DU CHERCHEUR

L'information qui figure sur ce formulaire de consentement et les réponses que j'ai données au·à la participant·e décrivent avec exactitude le projet.

Je m'engage à procéder à cette étude conformément aux bonnes pratiques des essais cliniques.

Je m'engage à ce que le participant à la recherche reçoive un exemplaire de ce formulaire de consentement.

Prénom, Nom ....

Signature ....

Date ....
